# Nanoscale mapping of carrier collection in single nanowire solar cells using X-ray beam induced current

**DOI:** 10.1107/S1600577518015229

**Published:** 2019-01-01

**Authors:** Lert Chayanun, Gaute Otnes, Andrea Troian, Susanna Hammarberg, Damien Salomon, Magnus T. Borgström, Jesper Wallentin

**Affiliations:** a Synchrotron Radiation Research and NanoLund, Lund University, Box 118, Lund 22100, Sweden; b Solid State Physics and NanoLund, Lund University, Box 118, Lund 22100, Sweden; c European Synchrotron Radiation Facility, 71 avenue des Martyrs, Grenoble 38043, France

**Keywords:** X-ray beam induced current, XBIC, nanowires, solar cells, internal quantum efficiency, IQE

## Abstract

Here it is demonstrated how nanofocused X-ray beam induced current (XBIC) can be used to quantitatively map the spatially dependent carrier collection probability within nanostructured solar cells. The photocurrent generated by a 50 nm-diameter X-ray beam was measured as a function of position, bias and flux in single p–i–n doped solar-cell nanowires. The signal gathered mostly from the middle segment decays exponentially toward the p- and n-segments, with a characteristic decay length that varies between 50 nm and 750 nm depending on the flux and the applied bias. The amplitude of the XBIC shows saturation at reverse bias, which indicates that most carriers are collected. At forward bias, the relevant condition for solar cells, the carrier collection is only efficient in a small region. Comparison with finite element modeling suggests that this is due to unintentional p-doping in the middle segment. It is expected that nanofocused XBIC could be used to investigate carrier collection in a wide range of nanostructured solar cells.

## Abstract 2

Nanofocused X-ray beam induced current (XBIC) is used to quantitatively map the spatially dependent carrier collection probability within single nanowires.

## 1. Introduction   

Nanostructures such as nanowires (Garnett *et al.*, 2011 ▸; Otnes & Borgström, 2017 ▸) and quantum dots (Nozik, 2002 ▸) are promising materials for next-generation photovoltaics because they absorb light efficiently while offering low material costs and unique mechanical properties (Fan *et al.*, 2009 ▸). In p–n junction based solar cells, light absorption generates non-equilibrium charge carriers, which are separated by the built-in electric field. Solar cells must collect these carriers with high efficiency. The charge collection in nanostructured solar cells can differ substantially from bulk cells of the same material due to a higher surface-to-volume ratio and increased current density (Schilinsky *et al.*, 2006 ▸; Kelzenberg *et al.*, 2008 ▸).

The probability of photogenerated electron–hole pairs to be collected is quantified by the charge collection probability (CCP), which is closely related to the internal quantum efficiency (IQE). While CCP and IQE are similar in principle, we mainly use CCP since it is more commonly used when discussing spatial variations in solar cells. These terms are distinct from the external quantum efficiency (EQE) which includes the efficiency of the generation process. Compared with light absorption and carrier generation, charge collection as reflected by IQE has been less studied in nanostructures. In solar cells, both the EQE and IQE are generally measured as a function of incident wavelength, with a large homogeneous beam. The CCP is affected by carrier lifetimes and local electric fields, which depend on doping and geometry. The CCP in nanostructured solar cells is expected to show spatial variation on a short length scale and the diffusion length of the minority carriers is often less than 100 nm (Gutsche *et al.*, 2012 ▸; Mohite *et al.*; 2012 ▸; Yang *et al.*, 2016 ▸). A suitable probe should therefore have a high spatial resolution with a sufficient penetration depth to probe the interior of complete solar cells.

Although theoretical modeling of the spatially dependent IQE or CCP in nanostructures has been reported (Christesen *et al.*, 2012 ▸; Yang *et al.*, 2016 ▸), experimental investigations suffer from the lack of a suitable method. The IQE of traditional solar cells is usually calculated from the measured EQE and reflectance, but this method has poor spatial resolution. Focused visible light can be locally employed in scanning photocurrent microscopy (SPCM) (Ahn *et al.*, 2007 ▸), but with a diffraction limit of several hundred nanometres its spatial resolution is insufficient for nanostructures. By contrast, in electron beam induced current (EBIC) measurements the electron beam can be easily focused to the nanometre scale (Hanoka, 1980 ▸), but the penetration depth can be too short to probe the interior of a complete solar cell.

As opposed to methods employing optical and electron beams, hard X-rays have very long penetration depths (Stuckelberger *et al.*, 2015 ▸), which makes them an ideal probe for *operando* and *in situ* studies (Vyvenko *et al.*, 2004 ▸). Modern X-ray optics allow focusing below 100 nm, with recent demonstrations showing sub-10 nm focusing (Mimura *et al.*, 2010 ▸; Döring *et al.*, 2013 ▸). In the X-ray beam induced current (XBIC) technique, a photocurrent is generated by X-rays (Vyvenko *et al.*, 2004 ▸; Villanova *et al.*, 2012 ▸; Wallentin *et al.*, 2014 ▸; Stuckelberger *et al.*, 2015 ▸; Johannes *et al.*, 2017 ▸). Spectrally resolved excitation with a tuneable X-ray energy has been used to excite specific elements (Johannes *et al.*, 2017 ▸; Chayanun *et al.*, 2018 ▸).

The XBIC process starts with photoelectric absorption of a primary X-ray photon with probability *p*_abs_, which creates a core hole and a photoelectron. The core hole is filled by an electron from an outer shell, generating a secondary photon (X-ray fluorescence, XRF) or an Auger electron. These secondary electrons and photons can in turn excite nearby atoms, creating an avalanche of electrons and photons. The average number of electron–hole pairs created per absorbed X-ray photon in a semiconductor is given by *η = *E*/∊*, where *E* is the photon energy and *∊* is the pair creation energy (Alig & Bloom, 1978 ▸). The X-ray absorption probability can be calculated by using the Beer–Lambert law. At the X-ray energy (17.5 keV) and sample thickness (180 nm) used in this study, we have η = 4122 and *p*_abs_ = 2.2 × 10^−3^ for bulk InP, and η = 3151 and *p*_abs_ = 2.7 × 10^−3^ for bulk In_0.56_Ga_0.44_P. Thus, more than one electron–hole pair are created per single photon event.

Once the excited electron–hole pairs have thermalized to the band edge, the CCP gives the probability for the charges to be collected and contribute to the XBIC signal. In this paper, we measure the CCP for the XBIC process, and a crucial point is how similar this is to the CCP for excitation with sunlight. To a first approximation, the drift, diffusion and recombination processes, and therefore the spatially dependent CCP, *S*(*x*, *y*, *z*), should be identical regardless of whether the primary excitation source was sunlight or X-ray. However, there are also important differences that will be discussed in connection with the modeling.

Since the nanowire solar cells studied here were created by a variation in doping along the axial direction *x*, we only consider the spatially dependent CCP along this axis, *S*(*x*). Furthermore, we can assume that η and *p*_abs_ are constant for scans along the axis of a single-material semiconductor nanowire with a constant diameter. The spatially dependent photocurrent *I*_ph_(*x*) can then be written as
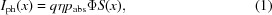
where *q* is the elementary charge and *Φ* is the incident photon flux. Thus, the measured photocurrent will be directly proportional to the CCP.

## 2. Results and discussion   

**Figure 1:**
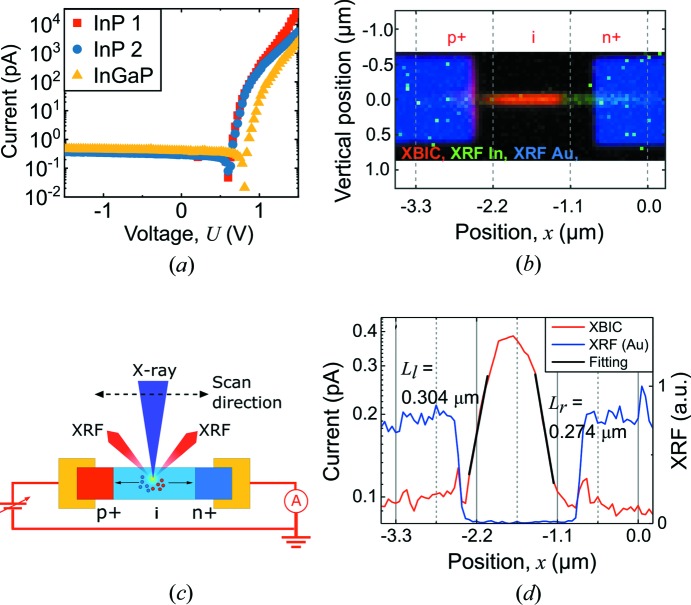
(*a*) Dark-current–voltage measurements. (*b*) Superpositioned image of the XBIC signals (red) and XRF signals for In (green) and Au (blue) from a two-dimensional scan of the InGaP nanowire. (*c*) Schematic of the XBIC experiment. (*d*) One-dimensional scan (50 nm step size) along the center of the nanowire showing the XBIC (red) and XRF signals (blue) in a semilogarithmic plot. The black lines are fits using exponential decay functions. The vertical gray lines correspond to the nominal length of each nanowire segment with the nanowire–Au particle interface at *x* = 0.

In this communication, we demonstrate how XBIC with nanofocused X-rays can be applied to quantitatively map the CCP with a spatial resolution of ∼50 nm. To this end, we investigated InP and In_0.56_Ga_0.44_P nanowires with axial p–i–n doping profiles, which were grown using the vapor–liquid–solid growth by use of Au seed particles (see supporting information). All three segments were 1.1 µm long and the diameter was 180 nm. The nanowires are being developed for solar cells with large arrays of as-grown vertical nanowires (Otnes *et al.*, 2018 ▸), but here we contacted single nanowires on a silicon nitride (Si_3_N_4_) membrane. Three devices, two with InP nanowires and one with an InGaP nanowire, were characterized. The dark-current–voltage measurements of these nanowires are shown in Fig. 1 ▸(*a*), yielding ideality factors of about 1.8 when fitting a single diode model at forward bias. The nanofocused X-ray beam [50 nm (H) × 60 nm (V)] at beamline ID-16B at the European Synchrotron Radiation Facility (ESRF), Grenoble, France, was used for excitation [Fig. 1 ▸(*c*)] (Martínez-Criado *et al.*, 2016 ▸).

We first present the spatially dependent XBIC, followed by systematic variations of X-ray flux and applied bias. Finally, we show how finite element modeling can be used to quantitatively explain the results.

### 2.1. Spatially dependent XBIC   

Initially we investigated the spatial resolution. The superpositioned XRF and XBIC signals measured at each position of the InGaP nanowire are displayed in Fig. 1 ▸(*b*). A similar result was obtained from the InP nanowire. The XRF signals from Au and In represent the metal contacts and the nanowire, respectively. The XBIC signal (red areas) was strongest in the middle of the nanowire, as expected. The image demonstrates the high-resolution maps obtained with the nanofocused X-ray probe.

A one-dimensional scan along the center of the nanowire is presented in Fig. 1 ▸(*d*). The vertical gray lines indicate the nominal positions of the segment junctions, which we obtained by using the XRF peak from the Au seed particle as a reference. In addition to the main peak, we observe weaker peaks at the edge of the metal contact on both sides, which we attribute to the indirect absorption in the nanowire of secondary photons or electrons emitted from the metal contact. We measured the devices with ground attached to either side but observed no difference, which is to be expected for direct photoemission from the contacts.

In an idealized p–i–n junction, the depletion region is defined by the middle segment, where the electric field is high and constant (Yang *et al.*, 2016 ▸). In our nanowires, the highest XBIC signal is indeed generated in the center of the middle segment, but it falls off towards the junctions on either side. The characteristic decay lengths, revealed by fitting exponential decay functions, *I ≃ * exp(*−x*/*L*), are *L*_l_ = 0.304 µm and *L*_r_ = 0.274 µm for the left and right slopes, respectively [Fig. 1 ▸(*d*)] (Gu *et al.*, 2006 ▸; Gutsche *et al.*, 2012 ▸; Chen *et al.*, 2015 ▸). The decay lengths cannot be directly identified by the minority carrier diffusion lengths, as we will demonstrate later.

### 2.2. X-ray flux variation XBIC   

**Figure 2:**
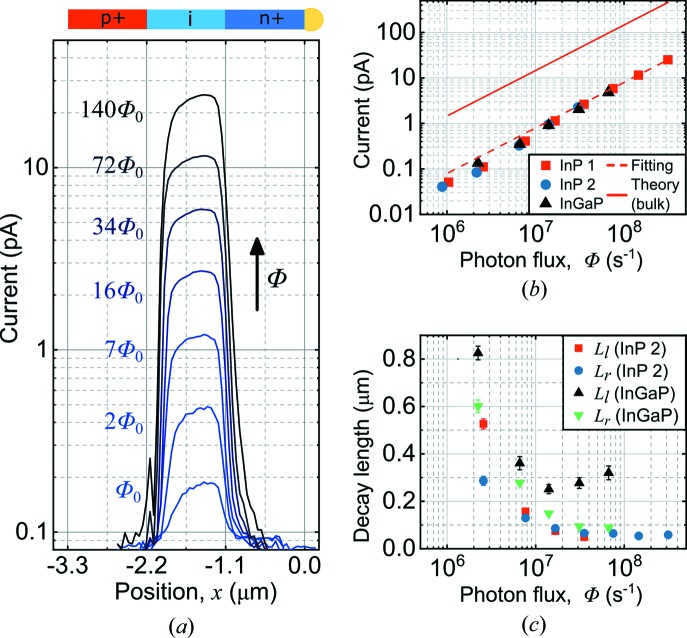
X-ray flux variation. (*a*) XBIC profiles at different X-ray fluxes, *Φ*, for the InP nanowire (Φ_0_ = 2.6 × 10^6^ s^−1^). The drawing nanowire indicates the nominal segment lengths. (*b*) Magnitude of the XBIC peak versus *Φ*. The solid line is calculated according to equation (1), and the dashed line is a linear fit to the measured data. (*c*) Fitted decay lengths, *L*_l_ and *L*_r_, for InP 2 and InGaP versus Φ. The error bars are a result of the decay length fitting (see Fig. S1).

Previous investigations of X-ray excitation of nanowire devices showed non-linear flux dependence, which was attributed to long-lived traps (Wallentin *et al.*, 2014 ▸; Chayanun *et al.*, 2018 ▸). We therefore varied the X-ray flux over two orders of magnitude at zero bias [Fig. 2 ▸(*a*)].

The magnitude of the XBIC peak was found to rise linearly with increasing X-ray photon flux [Fig. 2 ▸(*b*)], which suggests that the investigated nanowires are not affected by photogating and photodoping effects as previously observed in photoconductance measurements using X-ray (Wallentin *et al.*, 2014 ▸; Chayanun *et al.*, 2018 ▸) and UV (Soci *et al.*, 2007 ▸) excitation. However, the magnitude of the XBIC signal was only ∼5% of the calculated photocurrent [equation (1)]. We attribute this difference to higher losses of escaping secondary electrons and photons due to the small nanowire diameter (Stuckelberger *et al.*, 2017 ▸).

The excitation level in XBIC analysis of solar cells should ideally be similar to that under solar illumination. At the lowest X-ray flux of 2.6 × 10^6^ s^−1^, the carrier generation rate is 2.61 × 10^27^ m^−3^ s^−1^. The carrier generation in nanowire array solar cells under one sun illumination varies with incident wavelength, and increase from about 10^26^ m^−3^ s^−1^ at the base to over 10^28^ m^−3^ s^−1^ near the top facing the sun (Yang *et al.*, 2016 ▸). Thus, these XBIC investigations are carried out at the relevant excitation levels.

The qualitative shape of the XBIC peak remained similar as the photon flux increased, but we observed a broadening of the peak. Furthermore, the fitted decay lengths were found to decrease with increasing X-ray flux, from several hundred nanometres at a low flux down to about 50 nm at the highest examined flux [Fig. 2 ▸(*c*)]. The individual fittings of the decay lengths can be found in Fig. S1. The declining decay lengths of the nanowires suggest a decrease in carrier lifetime or mobility with increasing carrier density, possibly due to Auger recombination that is increased by carrier scattering.

### 2.3. Applied bias dependent XBIC   

**Figure 3:**
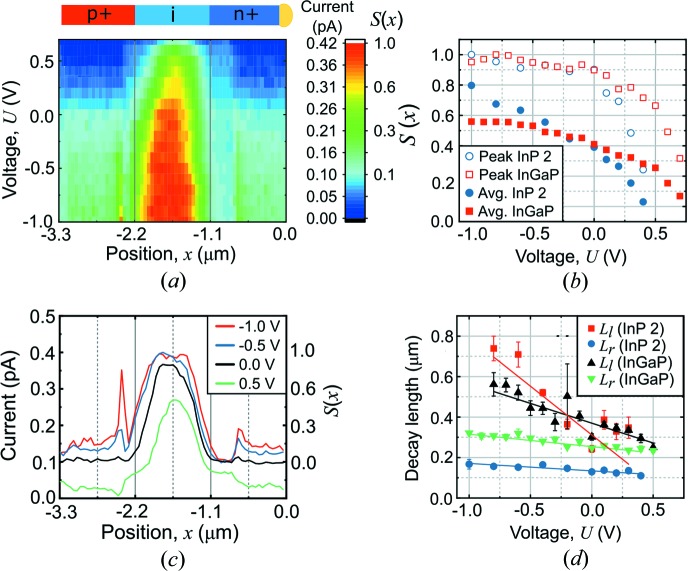
Applied bias voltage variation. (*a*) Map representing the XBIC and the spatial IQE, *S*(*x*), profiles across the InGaP nanowire as a function of applied bias. The drawing indicates the nominal segment lengths. (*b*) XBIC and *S*(*x*) profiles along the InGaP nanowire at a few selected biases. (*c*) The peak and the average *S*(*x*) within the middle segments versus applied bias. (*d*) The decay lengths *L*_l_ and *L*_r_ of InP2 and InGaP nanowires versus applied bias, including linear fits. The error bars are a result of the decay length fitting (Fig. S1).

The measurements presented so far were performed at zero bias, but solar cells are operated at forward bias in order to generate a useful external power. As we will show, the *S*(*x*) profiles are substantially different at forward bias. We investigated the XBIC as a function of applied biases at moderate photon flux, Φ = 6.5 × 10^6^ s^−1^ [Figs. 3 ▸(*a*) and 3(*b*)].

At reverse bias, the maximum XBIC increased marginally while the peak width increased substantially. The reverse bias increases the electric field in the depletion region, which gives the carriers less time to recombine before collection. The saturated current is a strong indication that the CCP is close to 100%, making the current limited by the generation process.

**Figure 4:**
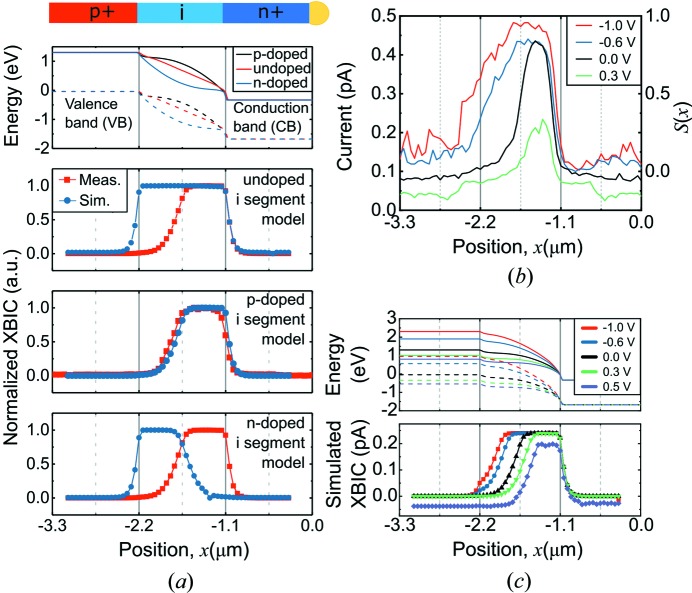
Simulated XBIC. (*a*) Normalized linear plots comparing the measured (red squares) and simulated XBIC (blue dots) of the InP nanowire for different doping types in the middle segment. The top plot represents the band structure for each case. Solid lines represent the conduction band and dashed lines represent the valence band. (*b*) XBIC and *S*(*x*) profiles along the center of the InP nanowire at different biases. (*c*) The simulated bias dependence of XBIC for the InP nanowire with a slightly p-doped middle segment.

The forward bias measurement shows that the maximum XBIC decreases with increasing bias [Figs. 3 ▸(*a*) and 3(*b*)]. The first obvious reason is that the dark current increases at forward bias, which can be observed as a downward shift of the entire *S*(*x*) curve. In addition, there is a reduction of the peak width and a slight shift towards the n-side, which is less intuitive. Even when subtracting the background current from the XBIC, the signal at forward bias is lower than at reverse bias. Hence, we conclude that the extent of the effective photo-collecting region is reduced at forward bias. A similar and stronger trend can be observed in the InP nanowire (described in Fig. 4 ▸).

An important challenge is to create a quantitative scale for the conversion of the measured XBIC into IQE or CCP, as recently discussed (Stuckelberger *et al.*, 2017 ▸). Compared with visible-light measurements, two factors complicate the analysis. First, the primary X-ray absorption, *p*_abs_, cannot be measured by reflectivity measurements. Second, the number of electron–hole pairs generated per X-ray photon, η, is much higher than one, and it can vary strongly with energy and position due to losses of secondary photons and electrons. Such losses, which in our case are evident in the flux-dependent measurements above, can in principle be modeled using Monte Carlo simulations (Stuckelberger *et al.*, 2017 ▸).

We employ a different approach for the quantification, using the reverse-bias measurements for calibration. The XBIC signal saturates at reverse bias, which means that no more carriers are collected even though the electric field increases. We make the assumption that the carrier collection is complete when the XBIC is saturated, such that *S*(*x*) = 1. Furthermore, the lower end of the scale, *S*(*x*) = 0, was defined at the background current which, owing to noise, raised the dark current to about 0.1 pA. With these two end points, it is possible to convert the measured XBIC into CCP (Fig. 3 ▸). These assumptions create an uncertainty in the absolute values for the calibration. However, the key ability of XBIC is to measure the relative spatial variation of the CCP with high spatial resolution, rather than absolute measurements.

The average CCP of the nanowire solar cell, assuming homogeneous excitation, can be estimated from the average *S*(*x*) within the middle segment*.* We compared the average *S*(*x*) (filled symbols) with the peak *S*(*x*) (unfilled symbols) as a function of applied bias, shown in Fig. 3 ▸(*c*). The peak *S*(*x*) plot has the same shape as the current–voltage curve (see supporting information), since *S*(*x*) is proportional to the XBIC according to equation (1). Even though the peak *S*(*x*) saturates at reverse bias, the average *S*(*x*) becomes greater with the increasing reverse bias because the width of the XBIC peak increases. The average *S*(*x*) drops at forward bias, especially in the InP nanowire, which means that the carrier collection efficiency of the solar cell is reduced.

To further understand how the forward bias affects the carrier transport, we investigated the bias dependence of the fitted decay lengths [Fig. 3 ▸(*d*)]. Although *L*_l_ of InP2 and InGaP decreases linearly with the bias, *L*_r_ is almost constant. Because the peak shifts towards the n-segment with increasing bias, the fitting range of the slope must be changed accordingly (see supporting information). Therefore, the range for *L*_l_ is shifted spatially, *i.e.* it is evaluated for different parts of the nanowire, while the range for *L*_r_ is rather constant.

Qualitatively, the decreased decay lengths at forward bias imply that the carriers can travel a shorter distance at forward bias before recombining. The increased bias weakens the built-in electric field of the depletion region, meaning that less photogenerated minority carriers drift to the end segments before recombining. In contrast, previous studies of p–n junction nanowires using electron-beam (Gutsche *et al.*, 2012 ▸) and laser excitation (Mohite *et al.*, 2012 ▸) showed decay lengths that were independent of bias.

### 2.4. Finite element study   

The results obtained by XBIC of our nanowires are significantly more complex than expected from an idealized p–i–n junction. To shed light on the underlying mechanism of the carrier collection, we therefore performed finite element modeling (Comsol Multiphysics version 5.2, COMSOL Inc., Stockholm, Sweden) similar to the reported EBIC modeling (Zhong *et al.*, 2016 ▸). The carrier generation was calculated from equation (1), adjusted for the 5% factor discussed above and literature values were used for the carrier mobility of the nanowires (Joyce *et al.*, 2013 ▸), while the carrier lifetimes were matched with the slope of the simulated and measured XBIC profiles.

In the first part of the simulations, we investigated the effect of non-intentional doping in the middle segment of the InP nanowire [Fig. 4 ▸(*a*)]. The first panel of Fig. 4 ▸(*a*) shows the band structure of the nanowire with three simulated doping types: slight p-doping (hole concentration, p = 2 × 10^15^ cm^−3^), undoped (intrinsic), and slight n-doping (electron concentration, n = 2 × 10^15^ cm^−3^). We found that with an intrinsic middle segment, corresponding to the idealized case, the simulated peak does not match the measured one because it extends too far towards the p-segment [second panel of Fig. 4 ▸(*a*)]. Instead, we achieved an almost perfect fitting when assuming a constant p-doping [third panel of Fig. 4 ▸(*a*)]. The p-doping effectively shifts the junction towards the n-segment. We obtained the opposite result in the slightly n-doped case [fourth panel of Fig. 4 ▸(*a*)]. Thus, the first part of the simulations suggests that the middle segment of the InP nanowire was unintentionally p-doped. A similar result was measured from an intentionally p-doped middle segment InP nanowire with EBIC (Otnes *et al.*, 2018 ▸).

We analyzed the XRF data from our devices in order to determine the p-doping (Zn) concentration in the nanowire, but we found that there was a strong background from the 10 nm-thick Zn layer used in the contacts. In a related study, we investigated isolated p–i–n doped InP nanowires using nano-XRF, and observed an unintentional Zn concentration of 5 × 10^17^ cm^−3^ in the middle segment (Troian *et al.*, 2018 ▸). The Zn concentration was attributed to memory effects from the highly doped p-segment which is grown first; this supports the assumption of unintentional p-doping. The result cannot be directly transferred to this study, however, as the growth conditions were different. Furthermore, XRF quantifies the Zn concentration, which can be significantly higher than the hole concentration that is relevant for the electrical properties.

The simulated band structures [Fig. 4 ▸(*a*)] also provide the distribution of the built-in electric field (Fig. S4), which separates the generated electrons and holes and therefore affects *S*(*x*). With p-doping in the middle segment, there is a high electric field close to the nominal i–n junction, instead of a homogeneous electric field over the middle segment as in the undoped case. The simulated potential is essentially constant in the left half of the middle segment, giving a low electric field and *S*(*x*). This leads to a shift of the XBIC peak toward the n-segment.

For the InGaP nanowire, the simulated XBIC with a constant doping in the middle segment could not be matched to the measurements (Fig. S3). We speculate that this appears due to a combination of gradually decaying p-doping memory effects and n-type surface pinning (Weert *et al.*, 2006 ▸; Han *et al.*, 2012 ▸), creating an effective p–n junction in the middle of the nominally i-segment (Jain *et al.*, 2014 ▸).

### 2.5. Bias-dependent simulation   

In the second part of the simulations, we used the p-doped middle segment model to replicate the bias-dependent XBIC [Figs. 4 ▸(*b*) and 4(*c*)]. The simulation exhibits the same main features as the measurements, in particular, the shift of the XBIC peak at forward bias. Note that the dark current gives a negative current away from the junction.

Bias-dependent 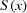 can also be understood from the simulated band structure in Fig. 4 ▸(*c*). With increasing forward bias, the potential drop is shifted toward the right part of the middle segment, which reduces the electric field and the width of the depletion region (supporting information). For this nanowire solar cell, around only half of the intended depletion region contributes to the photocurrent.

The simulations can also guide the interpretation of the observed decay lengths of the XBIC peak. We used the mobilities and the carrier lifetimes from the simulation to calculate the minority carrier diffusion lengths (*L* ^2^ = *kT*μτ/*q*) for the InP nanowire, which were *L*_e_ = 68 nm and *L*_h_ = 50 nm in the p- and n-segments, respectively. In contrast, the fitting of the measured and simulated XBIC peaks gives decay lengths of *L*_l_ = 148 nm and *L*_r_ = 59 nm, respectively. The decay length toward the p-segment (*L*_l_) is much longer than the calculated electron diffusion length (*L*_e_) which, together with strong bias dependence [Fig. 3 ▸(*d*)], demonstrates that it is affected by carrier drift. This strong bias-dependent *L*_e_ relates to the spatial variation of the built-in electric field, as illustrated by the simulation. In contrast, the n-side decay length (*L*_right_) is similar to the calculated hole diffusion length (*L*_h_) and is only marginally affected by the electric field [Fig. 3 ▸(*d*)].

## 3. Conclusions   

We have shown that XBIC with a nanofocused X-ray beam can be used to quantitatively investigate the spatially dependent CCP in single nanowires. The spatial resolution is sufficient to measure minority carrier diffusion lengths below 100 nm. We used this method to show that the investigated nanowire devices with p–i–n junctions deviate substantially from the ideal case, in particular under operationally relevant forward bias. Key aspects of the results could be explained using simulations.

Our investigations measure the CCP for XBIC excitation, and an important question is how similar this is to the CCP for sunlight excitation. A direct experimental comparison is challenging, but modeling and a careful analysis of the measurements can provide some indications. There are a two important differences between the two excitation modes.

First, the scanning XBIC measurements rely on creating carriers in a small segment of the nanowire, whereas the nanowire is more evenly excited in the operating solar cell. We note that the local excitation levels in this study are similar to those under one sun illumination, as discussed above. However, the localized excitation could still affect the drift, diffusion and recombination of charge carriers. We therefore simulated the Fermi levels and the carrier concentrations in the nanowire from both homogeneous and local excitation (Fig. S5). The simulations indicate that there are moderate differences at the relevant flux levels. Furthermore, the scans in Fig. 2 ▸ do not show any strong changes, neither quantitatively nor qualitatively, from a flux variation over two orders of magnitude. If the local excitation would lead to a strong distortion of the carrier collection probability, the distortion should become more severe at high fluxes, in contrast to the measurements. The XBIC excitation should ideally be performed as a small local perturbation of samples that are homogeneously excited by a sun simulator. While this is possible in principle, it was not available at the time of the experiment.

The second difference is that the synchrotron X-ray nanofocus is a pulsed source, with pulse length and period of about 0.1 and 3 ns, respectively. This is comparable with the recombination lifetimes of the charge carriers in the nanowires, which means that the carrier densities will exhibit time variation. Although it is possible to make time-resolved simulations of sub-nanosecond dynamics in nanowires with finite element modeling (Wallander & Wallentin, 2017 ▸), this is beyond the scope of the present study. The pulsed nature of synchrotron X-rays complicates the interpretation of the XBIC measurements, but also creates a future opportunity to study the dynamics of carrier collection with high temporal and spatial resolution.

Nanofocused XBIC can be used for a wide array of nanostructured solar cells, where the characteristic length scales are shorter than the diffraction limit of visible light. The long penetration depth of X-rays allows investigations of complete solar cells. This method can be combined with previously reported simulations and measurements of the spatial dependence of the absorption and carrier generation (Yang *et al.*, 2016 ▸). Future developments could use XBIC based on sub-10 nm X-ray focusing (Döring *et al.*, 2013 ▸) for improved spatial resolution which, in our case, could reveal any radial dependence of the carrier collection. XBIC could also be used to study carrier dynamics in other types of devices such as transistors and light-emitting diodes.

## 4. Related literature   

The following references are cited in the supporting information: Borgström *et al.* (2010 ▸); Bruce *et al.* (1990 ▸); Heurlin *et al.* (2015 ▸); Otnes *et al.* (2017 ▸).

## Supplementary Material

Supporting information. DOI: 10.1107/S1600577518015229/pp5131sup1.pdf
