# Supplementary material for: Nanoscale mapping of carrier collection in single nanowire solar cells using X-ray beam induced current
Source: J Synchrotron Radiat. 2019 Jan 1;26(Pt 1):102–8. doi: 10.1107/S1600577518015229 (PMC6337893; doi:10.1107/S1600577518015229)
Supplement: Supplementary file 1 [file s-26-00102-sup1.pdf]

JOURNAL OF SYNCHROTRON RADIATION

Volume 26 (2019)

Supporting information for article:

Nanoscale mapping of carrier collection in single nanowire solar cells using X-ray beam induced current

Lert Chayanun, Gaute Otnes, Andrea Troian, Susanna Hammarberg, Damien Salomon, Magnus T. Borgström and Jesper Wallentin

S1. Sample preparation

The investigated nanowires in this study are InP and $In_{0.56}Ga_{0.44}P$, which were synthesized by the vapor-liquid-solid (VLS) growth mechanism with Au seed particles. The growth temperature is 440 °C and the growth parameters are included in Tab. S1 and S2. The nanowire length was monitored in situ by use of optical reflectometry (Heurlin *et al.*, 2015, Otnes *et al.*, 2017). Hydrogen chloride (HCl) was applied during the syntheses to prevent a lateral growth (Borgström *et al.*, 2010).

For the nanowire device fabrication, The nanowires were transferred to suspended silicon nitride (Si_3N_4) membranes of 1 μm thickness (Silson), which were patterned with metal bond pads. Electron beam lithography (EBL) in combination with metal evaporation was used to defined metal contacts to individual nanowires. A Pd/Zn/Pd/Au (~10/10/10/170 nm) metal combination was used (Bruce *et al.*, 1990). The substrate was mounted and wedge bonded on a carrier chip, which is the platform of the device to connect to the other equipment.

The X-ray source was the ID16B beamline at the European synchrotron radiation facility (ESRF), France. The beam size was about 60 nm vertically and 50 nm horizontally. The X-ray was a pink beam ($dE/E \approx 10^{-2}$) with an energy of 17.5 keV. The flux of this X-ray beam was controlled by a set of metal filters. The XBIC and the XRF signals were simultaneously collected. The scanning was performed by moving the sample with piezo motors along the normal plane of the optical axis.

A voltage was applied to one end of the nanowire whereas the current was monitored by a current amplifier (Stanford research systems SR570) on the other end. For most measurements, we used a sensitivity of 10 or 100 pA/V, at which the amplifier has noise of 5 and 10 fA, respectively. We checked the noise and stability by making hundreds of repeated measurements at identical conditions, and we found that the variation was indeed 5-10 fA with the X-ray beam on and a few fA with the beam off. We also checked the sensitivity to ambient light. Since the microscope lamp gave a strong response it was turned off, while the ambient room light was found not to affect the measurements.

Table S1 Parameters used for the InP nanowire growth.

Segment	Time (s)	Molar fractions						Length after segment (nm)
		TMIn	TMGa	PH3	HCl	DEZn	TESn	
p	536	5.9E-5	-	6.9E-3	4.6E-5	4.6E-5	-	1210
i	792	7.4E-5	-	6.9E-3	4.6E-5	-	-	2400
n	1161	5.9E-5 (increased to 7.4E-5 during last 5 min)	-	6.9E-3	4.6E-5	-	4.3E-5	3430

Table S2 Parameters used for the InGaP nanowire growth.

Segment	Time (s)	Molar fractions						Length after segment (nm)
		TMIn	TMGa	PH3	HCl	DEZn	TESn	
InP stub	60	5.9E-5	-	6.9E-3	4.6E-5 (No HCl first for 15 sec)	-	-	~150
p	417	5.2E-5	3.9E-4	6.9E-3	5.4E-5	8.3E-5	-	1220
i	1202	2.7E-5	1.37E-3	5.4E-3	5.4E-5	-	-	2340
n	1417	2.7E-5	1.37E-3	5.4E-3	5.4E-5	-	4.4E-5	3495

S2. Simulation with Comsol

Comsol Multiphysics (version 5.2, COMSOL AB, Stockholm,Sweden) is a commercial software that can solve the Poisson's equation for electrostatic potential, and the continuity transport equations for the charge carrier concentrations. Therefore, it is a robust tool to study the band structure and the charge carrier transport within semiconductors. We assumed radial symmetry within the nanowires, and used the 1D analysis system with the Semiconductor module. The simulation model of the nanowire is a single line in which the junctions and metal contacts were assign to points in the nanowire. The evenly photoexcited area was defined in a region between two positions (60 nm apart), and then these positions were shifted along the nanowire line to simulate the scanning of the photoexcitation over the nanowire. All of the parameters used in this simulation are presented in Tab. S3.

Table S3 Parameters used for the XBIC simulation with Comsol. * The doping types is varied between p-and n-doped with the same doping concentration.

Segment	parameter	Value (InP)	Value (InGaP)
p	Doping (cm^{-3})	$2x10^{18}$	$2x10^{18}$
	τ_n (ns)	0.001	0.008
	τ_p (ns)	0.7	0.8
i	Doping* (cm^{-3})	$2x10^{15}$	$5x10^{15}$
	τ_n (ns)	0.002 (*p*-doped)<br>1.0 (*n*-doped)<br>1.0 (intrinsic)	0.05 (*p*-doped)<br>1.0 (*n*-doped)<br>1.0 (intrinsic)
	τ_p (ns)	1.0 (*p*-doped)<br>0.01 (*n*-doped)<br>1.0 (intrinsic)	1.0 (*p*-doped)<br>0.05 (*n*-doped)<br>0.95 (intrinsic)
n	Doping (cm^{-3})	$2x10^{19}$	$2x10^{19}$
	τ_n (ns)	0.3	0.3
	τ_p (ns)	0.005	0.005
	μ_n (cm^2/Vs)	900	200
	μ_p (cm^2/Vs)	200	100
	Carrier generation rate ($m^{-3}s^{-1}$)	$1x10^{27}$	$1x10^{27}$

S3. Decay fit of the XBIC peaks

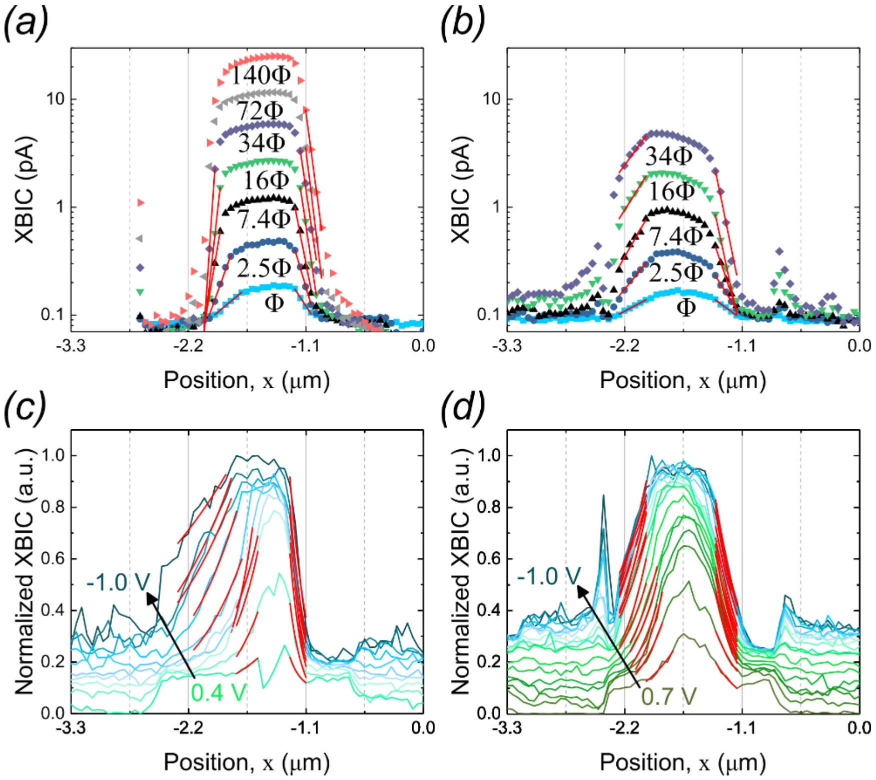

Figure S1 (a) and (b) Dependence of XBIC on the flux variation ($\Phi \approx 2.6\times10^{6}$ s^{-1}) including decay fits (red) for the InP (a) and InGaP nanowires (b). (c) and (d) The bias dependent XBIC with the decay fits (red) for the InP (c) and InGaP nanowires (d). The bias range is from -1.0 to 0.4 V for the InP nanowire and -1.0 to 0.7 V for the InGaP nanowire. The bias was increased in steps of 0.1 V except for the InP measurements at reverse bias, where it was varied in the steps of 0.2 V.

S4. Maximum XBIC vs. the applied biases

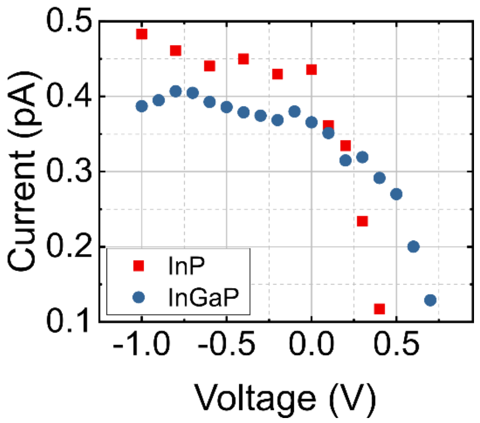

Figure S2 The maximum measured value of the XBIC vs. bias voltage from InP (red) and InGaP (blue) devices.

S5. Simulation of the InP and InGaP device

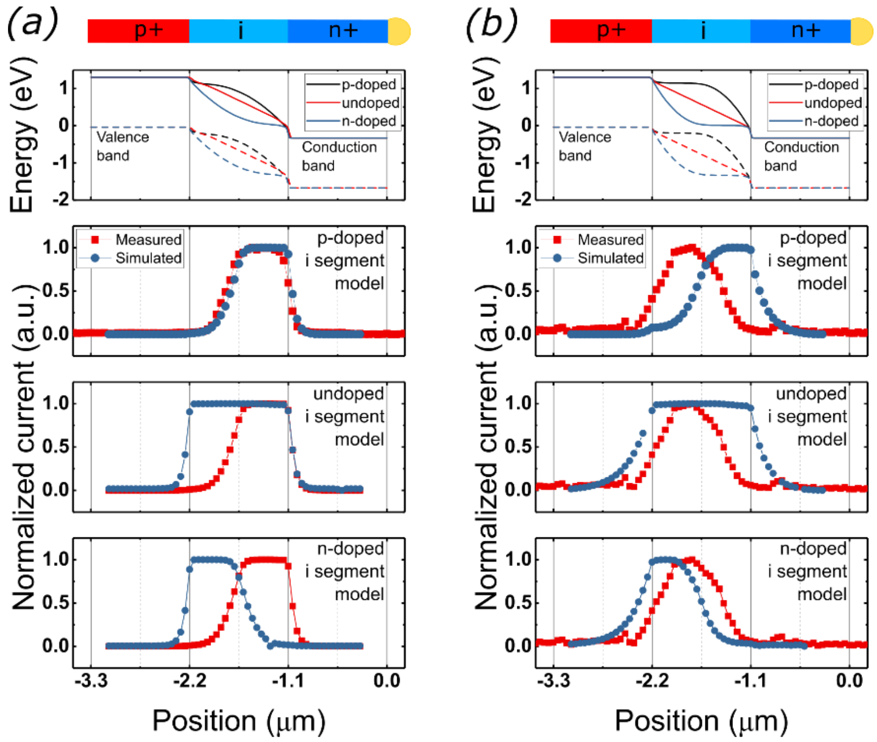

Figure S3 The plots in the first row represents the band structures for slightly p-doped (black), undoped (red), and slightly n-doped (blue) middle segments for InP (a) and InGaP nanowire (b). The following rows represents normalized linear plot comparing the measured (red squares) and simulated XBIC profiles (blue dots) for different doping type of the middle segment. The simulated result of the InP nanowire is displayed here again for easy comparison with the InGaP nanowire.

S6. Electric field distribution

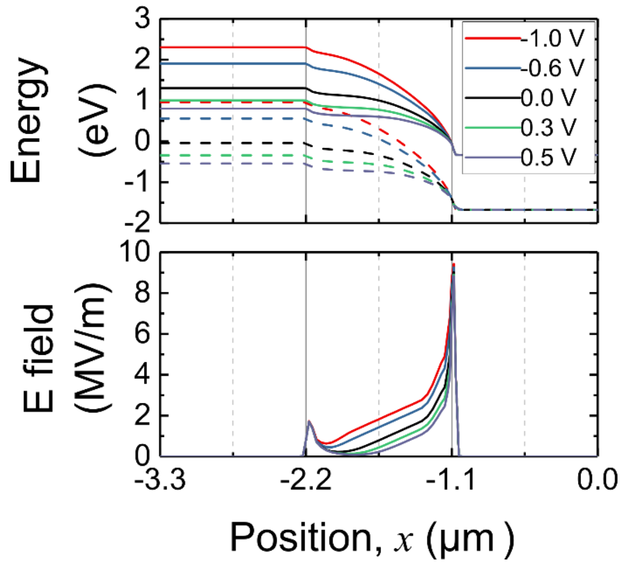

Figure S4 The band structure (top) and the electric field distribution (bottom) obtained from the simulations at different biases.

S7. Simulated band structure and carrier concentration with the various excitations

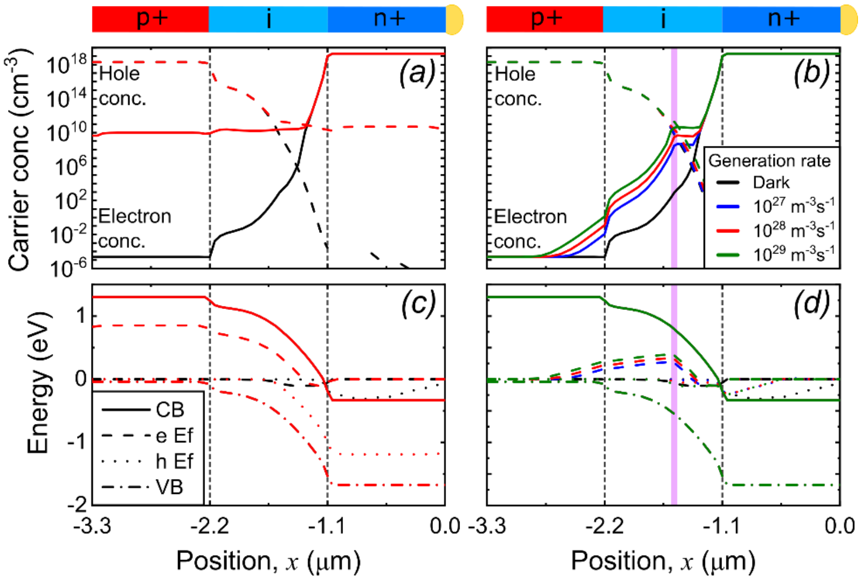

Figure S5 Electron and hole concentrations (solid and dashed line, respectively) along the nanowire (a) under the dark condition (black) and the homogeneous excitation (red) over the entire nanowire device and (b) with the local excitation (thick vertical purple line). The various generation rate in (a) and (b) is represented by the different colour as shown in the legend of (b). The simulated band structure in (c) and (d) correspond to the excitations as shown in (a) and (b), respectively. These plots include conduction band (solid line), Fermi level of electrons (dashed line), Fermi level of holes (dotted line) and valence band (dash dotted line).

References

Borgström, M. T., Wallentin, J., Trägårdh, J., Ramvall, P., Ek, M., Wallenberg, L. R., Samuelson, L. & Deppert, K. (2010). *Nano Research* **3**, 264-270.

Bruce, R., Clark, D. & Eicher, S. (1990). *Journal of Electronic Materials* **19**, 225-229.

Heurlin, M., Anttu, N., Camus, C., Samuelson, L. & Borgström, M. T. (2015). *Nano Letters* **15**, 3597-3602.

Otnes, G., Heurlin, M., Zeng, X. & Borgström, M. T. (2017). *Nano Letters* **17**, 702-707.
